# Assessment of Drain Water Used for Irrigation in the Delhi Region

**DOI:** 10.5696/2156-9614-10.26.200610

**Published:** 2020-05-28

**Authors:** Deepak Gola, Arghya Bhattacharya, Priyadarshini Dey, Anushree Malik, Shaikh Ziauddin Ahammad

**Affiliations:** 1 Applied Microbiology Lab, Centre for Rural Development and Technology, Indian Institute of Technology, Hauz Khas, Delhi, India; 2 Department of Biotechnology, Noida Institute of Engineering and Technology, Uttar Pradesh; 3 Department of Biochemical Engineering and Biotechnology, Indian Institute of Technology, Hauz Khas, Delhi, India

**Keywords:** wastewater, heavy metal, irrigation, health risk

## Abstract

**Background.:**

Industries such as electroplating, mining and battery production are major sources of heavy metal-rich waste entering nearby water bodies. Irrigation with heavy metal contaminated water can deteriorate soil quality as well as agricultural produce and have further toxic effects on human health.

**Objectives.:**

The objective of the present study was to estimate the concentration of hazardous heavy metals such as chromium (Cr), cadmium (Cd), copper (Cu), nickel (Ni), zinc (Zn) and lead (Pb), as well as physico-chemical variables (pH, electrical conductivity, total dissolved solids, chemical oxygen demand and dissolved oxygen) at sampling locations along the Najafgarh and Loha mandi drains in Delhi, National Capital Region, India.

**Methods.:**

The present study evaluated the quality of wastewater from the Najafgarh and Loha mandi drains, which are used for irrigational purposes in the Delhi region. Drain water quality was monitored for a period of 2 years for physico-chemical variables (pH, chemical oxygen demand, electrical conductivity and dissolved oxygen) as well as heavy metal concentrations (Cr, Cu, Cd, Zn, Ni and Pb). The two-year monitoring period (July 2012–March 2014) was chosen to represent three seasons: pre-monsoon, monsoon, and post-monsoon.

**Results.:**

Varied concentrations of multiple heavy metals were found due to the extensive discharge of untreated industrial effluents into the drain water. Punjabi Bagh of Najafgarh drain was the most contaminated sampling site with the maximum concentration of Zn (12.040 ± 0.361 mg L^−1^), followed by Cr (2.436 ± 0.073mg L^−1^) and Cu (2.617 ± 0.078 mg L^−1^).

**Conclusions.:**

Consumption of heavy metal-contaminated agricultural products can cause deleterious human health effects, leading to further health problems. The presence of multi-heavy metal ions above the Food and Agriculture Organization of the United Nations (FAO) permissible limits indicated that drain water was not suitable for irrigational purposes, and adequate measures are required to remove the heavy metal load from drain water.

**Competing Interests.:**

The authors declare no competing financial interests.

## Introduction

The agriculture sector accounts for more than 70% of global water usage.^1^ However, the increasing demand for water from the energy and municipal industries in peri-urban and urban areas decreases the availability of fresh groundwater for agriculture.^2–5^ A survey across different government agencies predicted a large increase in water demand by 2050 (in total 1447 km^3^) by each sector, with the agricultural sector continuing to demonstrate the highest demand compared to other sectors.^6^ At the same time, inequality in supply and demand is also projected due to multiple factors such as population explosion, urbanization and environmental (climate) changes, which can worsen the situation of freshwater availability.^7–9^ The development of bores and wells by the Indian government at subsidized cost for irrigational purposes has severely depleted the water table.^10^ Severe water stress at present and in the near future highlights the need for alternatives, such as wastewater, to fulfill irrigational requirements.^2^ The use of municipal and industrial wastewater for irrigation whether untreated or treated, has been widely practiced in urban and peri-urban areas in India and many other countries.^11,12^ Studies have reported the presence of beneficial nutrients in wastewater, but the volume of available wastewater is very low.^12,13^ The biggest challenge in using wastewater for irrigation is that along with beneficial nutrients, crops are also exposed to toxic contaminants.^7,14^ The occurrence of toxic contaminants like heavy metals in wastewater are the result of metal processing and polishing industries, as partially treated or treated effluents are directly discharged into the waste stream.^2,15^ There are close to 100,000 small-scale industries located in Delhi's residential and non-conforming areas, which largely contribute to air and water pollution, due to the lack of a proper waste treatment facility and wastewater discharged directly into nearby drains that later join the Yamuna River.^16^

Untreated or partially treated wastewater contains a wide range of heavy metals that can contaminate soil as well as crops/vegetables, leading to neurological, respiratory, immunological, renal or carcinogenic problems for consumers.^17,18^ A recent report indicated the presence of heavy metal ions in vegetable and fruit cultivated using contaminated wastewater and water from the Yamuna River and nearby drains.^19^ This drain network system carries wastewater from nearby human and industrial activities, such as domestic wastewater, industrial wastewater, ground and storm water runoff. Hence the objective of the present study was to estimate the concentration of hazardous heavy metals like chromium (Cr), cadmium (Cd), copper (Cu), nickel (Ni), zinc (Zn) and lead (Pb), as well as physico-chemical variables such as pH, electrical conductivity, total dissolved solids, chemical oxygen demand and dissolved oxygen at sampling sites along the Najafgarh and Loha mandi drains in Delhi, National Capital Region, India.

**Abbreviations**
FAO: Food and Agriculture Organization of the United Nations

## Methods

**Figure 1:**
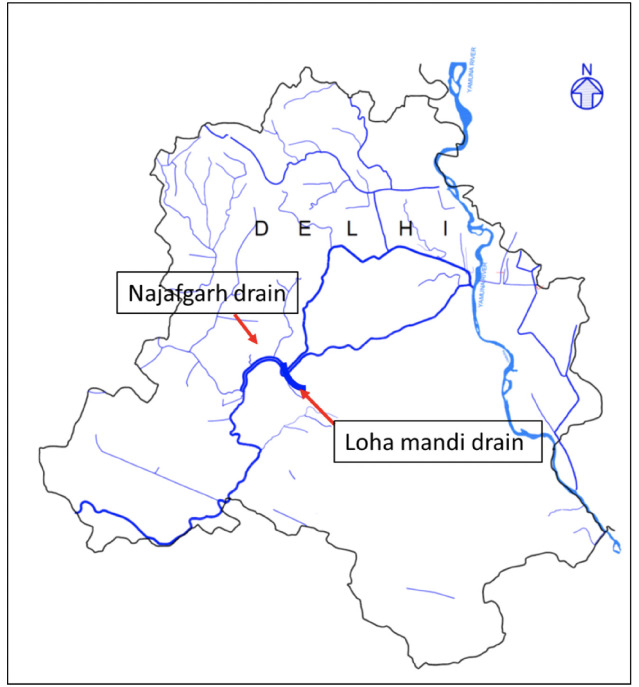
Location of Najafgarh and Loha mandi drain within the National Capital Territory of Delhi

**Figure 2:**
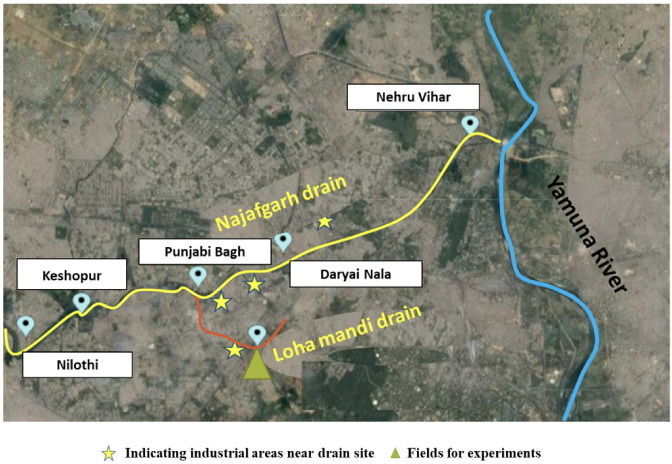
Location of sampling sites along the Najafgarh and Loha mandi drains (Map was created with the help of Mapcustomizer)

Two major drains in Delhi, (officially the National Capital Territory of Delhi), the Najafgarh and Loha mandi drains, were selected for sampling and water quality analysis as a large number of small scale industrial operations (battery, paints, electroplating, alloy, pickling, printing, chemical, plastic, galvanization, etc.) in Delhi are concentrated around these drains.^7^ The location of these drains on Delhi map is shown in Figure 1. The Najafgarh drain is the largest of all the surface drains joining the river in the National Capital Territory. Its total catchment area is around 374 km^2^ and it discharges around 2000 million L wastewater (domestic and industrial) per day to the Yamuna River near the Wazirabad section.^20^ A large volume of food waste, fecal matter and other forms of organic and inorganic wastes are often dumped in the Najafgarh drain. The five sampling sites selected over the stretch of the Najafgarh drain included Nilothi, Keshopur, Punjabi Bagh, Daryai Nala, and Nehru Vihar, all densely populated slums. The locations of sampling sites are shown in Figure 2. The second drain selected for sampling was the Loha mandi drain *(Figure 2).* This drain has a total catchment area of 6.4 km^2^. It joins the Najafgarh drain at Sudershan Park after passing through the Indian Council of Agricultural Research (ICAR), Pusa campus, Delhi, India. This site is close to Naraina Vihar (well known for steel/iron industries and markets) and is expected to be contaminated with metals.

### Collection of water samples and characterization

Wastewater samples were collected from a depth of 150–300 mm in triplicates in 5 L sampling bottles (polypropylene, Borosil) from the five locations along the Najafgarh drain and one location along the Loha mandi drain. Bottles were washed with dilute chromic acid and thoroughly rinsed with tap water prior to wastewater sampling. All the collected wastewater samples were filtered using cotton cloth to remove any large suspended particles/debris and acidified with nitric acid (36%) before preservation at 4°C. The heavy metal analysis was performed within a period of 24 hours. Sample collection was done over a period of two years (2012–2014) in three seasons: (a) post-monsoon (September–December); (b) pre-monsoon (January–June), and (c) monsoon (July–August).

Physicochemical variables pH, total dissolved solids, electrical conductivity and dissolved oxygen were measured at the sampling sites using portable probes. pH was measured with a portable ion meter (Hanna Instruments HI98121) while total dissolved solids was measured by Eutech CyberScan PC510. Dissolved oxygen was estimated using Eutech CyberScan DO 110. Chemical oxygen demand and heavy metal concentrations were measured after transporting samples to the lab. Samples were digested (165–170°C for 10 minutes for reaction) with acid for heavy metal analysis using 4200 MP-AES (Agilent Technologies).

### Reagent and standard preparation

Analytical grade chemicals (Central Drug House, Merck Group and Sisco Research Laboratories) were used in the above analyses without any further purification. Deionized ultrapure water (RIONS, D-Ultra) was used to prepare all the reagents as well as calibration standards. The heavy metal standards for the calibration were prepared from 1000 mg L^−1^ stock solution of each heavy metal via dilution with deionized water.

### Chemical oxygen demand

For chemical oxygen demand analysis, 10 mL wastewater samples were subjected to centrifugation at 8000 g for 10 minutes and supernatant was obtained. Supernatant sample chemical oxygen demand was estimated by oxidizing the fixed amount of sample volume via potassium dichromate (HACH method 8000) resulting in a reduction of chromium from the hexavalent ion to the trivalent ion. All the samples were digested for two hours at 150°C ± 5°C in a digester (Hach Digital Reactor DRB200). After sample digestion, samples were centrifuged to remove insoluble suspended matter. Further, the blank sample was prepared in parallel using doubled distilled water and digested with conditions as mentioned above. The readings of each sample were taken using a Hach multi-meter (DR/890 colorimeter).

### Heavy metal concentration in wastewater

Heavy metal ion (Cr, Cu, Cd, Ni, Zn, and Pb) concentrations in wastewater samples were determined after acid digestion with nitric acid (15 N and 65% pure Emsure, Merck) in the ratio 9 (sample):1 (acid) in a microwave digester (Multiwave Pro, Anton Paar) using standard protocol (150 ± 4°C within 10 minutes and 165–170°C ± 5°C for 10 minutes for digestion reaction).^21^ The digested solution was centrifuged and screened through Whatman number 1 film paper. The filtrate was collected in separate tubes and heavy metal concentrations were quantified using 4200 MPAES (Agilent Technologies). The concentrations of other elements in *Loha mandi* drain wastewater was analyzed by ICP-OES (Inductively Coupled plasma-optical emission spectrometry, 5100-Agilent), using the same digestion protocol.

### Statistical analysis

All analyses were conducted in triplicates and the results are expressed in terms of mean ± SD calculated using XLSTAT 2014. Multivariate analysis was performed on the data using principal component analysis on wastewater collected from the Najafgarh drain.

## Results

All physico-chemical variable results were compared with the Food and Agricultural Organization of the United Nations (FAO) permissible limits.^2^ Table 1 indicates the average values of physico-chemical variables of wastewater collected from the sampling sites.

**Table 1:** Average Values of Physico-Chemical Variables in Wastewater

	**Average value of physico-chemical variables**				

**Sampling sites**	**TDS (mg L^−1^)**	**pH**	**DO (mg L^−1^)**	**COD (mg L^−1^)**	**EC (μSiemens)**
Najafgarh drain	357	7.60	4.74	68.9	479.05
Nilothi					
Keshopur	293	7.42	4.66	137.4	390.2
Punjabi Bagh	324	7.52	4.8	192.4	440.2
Daryai Nala	249	7.32	4.38	244.1	342.4
Nehru Vihar	302	7.38	6.42	209.4	410
Loha mandi	**800**	7.7	7.9	**297.8**	1379
*Permissible limits (FAO)^22^*	*450*	*6.5–8.4*	**-**	*250*	*100–2250*

The concentration of physico-chemical parameters which lies above permissible limits (FAO) are indicated with bold numeric values.

Abbreviations: TDS, total dissolved solids; DO, dissolved oxygen; COD, chemical oxygen demand; EC, electrical conductivity.

### pH values

The optimum range of pH according to the FAO standards for irrigation is 6.5–8.4.^22^ The pH values (7.32–7.60) were slightly alkaline at an average temperature of 27°C.

### Total dissolved solids

Values for total dissolved solids at the various sampling sites of the Najafgarh drain ranged on the lower side (249–357 mg L^−1^) at an average temperature of 27°C. The range of total dissolved solids values was well within the permissible limits (450 mg L^−1^). The average total dissolved solids value at the Loha mandi drain site (800 mg L^−1^) was above FAO permissible limits for irrigation.^22^

### Electrical conductivity

Electrical conductivity, which is a measure of salinity, was found to be in the range of 342.4–479.05 μSiemens at the Najafgarh sites and 1379 μSiemens at the Loha mandi drain at an average temperature of 27°C, which is within the stipulated range of 100–2250 μSiemens as per FAO limits for irrigation.^22^

### Chemical oxygen demand

Chemical oxygen demand values are useful for identifying toxic conditions as well as the presence of biologically resistant contaminants or matter in wastewater. The chemical oxygen demand varied from 68.9 mg L^−1^ at Nilothi to 244.1 mg L^−1^ at Daryai Nala along the Najafgarh drain. The chemical oxygen demand values were within the range of the dischargeable limit of 250 mg L^−1^, whereas at the Loha mandi drain the average chemical oxygen demand value was found to be above the permissible limits (297.8 mg L^−1^). High chemical oxygen demand in wastewater could disturb the oxygen transfer rate to agricultural soil, negatively impacting the growth of plants/crops.^7^

### Assessment of heavy metal concentrations in collected wastewater

Guidelines have been issued by various governing bodies and agencies describing the threshold limit of contaminants or pollutants, including heavy metals, in wastewater used for irrigation. However, in India there are no such guidelines and recommendations pertaining to permissible threshold limits of heavy metals in irrigation water. Hence the FAO guidelines were considered for the present study.^22^ The maximum and minimum concentrations of heavy metals measured in wastewater collected from both drains along with descriptive statistics are presented in Table 2.

**Table 2:** Heavy Metal Concentrations in Wastewater from the Najafgarh and Loha Mandi Drains

**Variable**		**Mean**	**Median**	**SD**	**Minimum**	**Maximum**
Najafgarh drain	Cu	0.168	0.010	0.401	0	2.617
	Cr	0.139	0	0.377	0	2.436
	Pb	0.161	0.034	0.301	0	1.579
	Zn	0.438	0.070	1.690	0	12.40
	Cd	0	0	0.010	0	0.075
	Ni	0.043	0	0.102	0	0.596
Loha mandi drain	Cu	0.155	0.037	0.121	0	0.580
	Cr	0.012	0	0.028	0	0.090
	Pb	0.074	0.016	0.146	0	0.470
	Zn	0.263	0.072	0.316	0	1.110
	Cd	0.002	0	0.006	0	0.020
	Ni	0.053	0	0.089	0	0.220

### Copper in the Najafgarh drain

According to FAO permissible limits, the concentration of Cu in wastewater should not exceed 0.2 mg L^−1^.^22^ The concentration of Cu across all the selected sites was very high in September 2012 and October 2013 *(Table 3).* Punjabi Bagh was found to be the most contaminated site along the Najafgarh drain, followed by Nehru Vihar and Daryai Nala, whereas the Nilothi and Keshopur sites were less contaminated compared to the other three sites. The maximum concentration of Cu was found to be 2.61 mg L^−1^ in the month of February 2013 at Punjabi Bagh.

**Table 3:** Copper Concentrations in Wastewater at Sampling Sites Along Najafgarh Drain

	**Copper**									

**Sampling sites**	**Jul. 2012**	**Sep. 2012**	**Dec. 2012**	**Feb. 2013**	**Apr. 2013**	**Jun. 2013**	**Aug. 2013**	**Oct. 2013**	**Jan. 2014**	**Mar. 2014**
Nilothi	ND	**0.301±0.009**	ND	ND	ND	0.018 ±0.000	0.119 ±0.004	**0.259 ±0.021**	ND	0.006 ±0.001
Keshopur	ND	**0.319±0.009**	0.010±0.000	ND	ND	0.170±0.005	0.097±0.003	**0.342±0.05**	ND	0.100±0.004
Punjabi Bagh	ND	**0.289±0.008**	ND	**2.617±0.078**	0.015±0.002	0.09±0.002	**0.860±0.026**	**0.436±0.004**	ND	ND
Daryai Nala	ND	**0.327 ±0.009**	ND	**0.289 ±0.008**	ND	0.110 ±0.003	0.029 ±0.009	**0.249 ±0.009**	ND	ND
Nehru Vihar	ND	**0.327±0.009**	ND	0.046±0.001	ND	0.080±0.002	**0.276±0.008**	**0.643±0.010**	ND	0.002±0.000

Limit for irrigation according to FAO (0.2 mg L^−1^).^22^

The concentration of heavy metals which lies above permissible limits (FAO) arc indicated with bold numeric values.^22^

Abbreviation: ND, not detected.

### Chromium in the Najafgarh drain

The FAO permissible limit of Cr in wastewater for irrigation purposes is 0.01 mg L^−1^.^22^ Although the occurrence of Cr varied across sampling seasons, it was found to be above permissible limits at all the sampling sites along the Najafgarh drain in one or more seasons *(Table 4).* Punjabi Bagh and Darayi Nala were more highly contaminated compared to other sampling sites along the Najafgarh drain.

**Table 4:** Chromium Concentrations in Wastewater at Sampling Sites Along the Najafgarh Drain

	**Chromium**									

**Sampling Sites**	**Jul 2012**	**Sep 2012**	**Dec 2012**	**Feb 2013**	**Apr 2013**	**Jun 2013**	**Aug 2013**	**Oct 2013**	**Jan 2014**	**Mar 2014**
Nilothi	ND	ND	ND	ND	ND	ND	**0.119 ±0.004**	**0.259 ±0.021**	ND	**0.103 ±0.022**
Keshopur	ND	ND	ND	**0.080 ±0.002**	0.002 ±0.004	ND	**0.097 ±0.003**	**0.342 ±0.05**	ND	ND
Punjabi Bagh	ND	ND	**0.083 ±0.002**	**2.436 ±0.073**	0.034 ±0.006	ND	**0.860 ±0.026**	**0.436 ±0.004**	ND	ND
Daryai Nala	ND	ND	**0.065 ±0.002**	**0.125 ±0.003**	ND	ND	**0.029 ±0.009**	**0.249 ±0.009**	ND	**0.056 ±0.001**
Nehru Vihar	ND	ND	ND	**0.271 ±0.008**	ND	**0.300 ±0.000**	**0.276 ±0.008**	**0.643 ±0.010**	ND	ND

Limit for irrigation according to FAO (0.01 mg L^−1^).^22^

The concentration of heavy metals which lies above permissible limits (FAO) are indicated with bold numeric values.

Abbreviation: ND, not detected.

### Lead in the Najafgarh drain

The concentration of Pb was found to be below permissible limits at all sampling sites as per FAO guidelines (5 mg L^−1^).^22^ However, Pb was present consistently throughout the sampling period at all sites at very low levels ranging from 0–1.5 mg L^−1^ *(Table 5).*

**Table 5:** Lead Concentrations in Wastewater at Sampling Sites Along the Najafgarh Drain

	**Lead**									

**Sampling Sites**	**Jul. 2012**	**Sep. 2012**	**Dec. 2012**	**Feb. 2013**	**Apr. 2013**	**Jun. 2013**	**Aug. 2013**	**Oct. 2013**	**Jan. 2014**	**Mar. 2014**
Nilothi	ND	ND	0.024 ±0.000	ND	0.024 ±0.000	0.056 ±0.000	0.004 ±0.000	0.267 ±0.008	0.206 ±0.006	0.023 ±0.002
Keshopur	ND	ND	ND	0.007 ±0.000	ND	0.110 ±0.003	0.112 ±0.003	0.420 ±0.013	0.622 ±0.003	0.123 ±0.006
Punjabi Bagh	ND	ND	0.034 ±0.001	0.688 ± 0.020	0.034 ±0.001	0.111 ±0.003	0.112 ±0.0033	0.369 ±0.011	0.455 ±0.090	0.056 ±0.008
Daryai Nala	ND	ND	0.024 ±0.000	0.082 ±0.002	0.024 ±0.000	0.070 ±0.002	0.068 ±0.002	0.522 ±0.016	1.163 ±0.008	0.067 ±0.000
Nehru Vihar	ND	ND	0.062 ±0.001	0.033 ±0.001	0.062 ±0.001	ND	ND	0.216 ±0.006	1.579 ±0.021	0.102 ±0.001

Limit for irrigation according to FAO (5 mg L^−1^).^22^

### Zinc in the Najafgarh drain

According to FAO guidelines, the threshold value of Zn is 2 mg L^−1^ in wastewater used for irrigational purposes.^1^ Zinc was detected in all samples collected, but at low levels. The presence of Zn in wastewater was detected persistently from June 2013 to March 2014 *(Table 6).* Zinc was detected above the permissible limit (12.04 mg L^−1^) in wastewater collected from Punjabi Bagh in February 2013.

**Table 6:** Zinc Concentrations in Wastewater at Sampling Sites Along the Najafgarh Drain

	**Zinc**									

**Sampling Sites**	**Jul. 2012**	**Sep. 2012**	**Dec. 2012**	**Feb. 2013**	**Apr. 2013**	**Jun. 2013**	**Aug. 2013**	**Oct. 2013**	**Jan. 2014**	**Mar. 2014**
Nilothi	ND	0.872±0.026	ND	ND	0.10 ±0.007	0.220 ±0.006	0.021 ±0.001	0.235 ±0.003	0.30 ±0.004	0.018 ±0.012
Keshopur	ND	0.459 ±0.013	ND	ND	0.031 ±0.010	0.300 ±0.009	0.011 ±0.001	0.246 ±0.005	0.42 ±0.005	0.023 ±0.010
Punjabi Bagh	ND	0.241 ±0.007	ND	**12.040 ±0.361**	0.008 ±0.005	0.980 ± 0.029	0.041 ±0.001	0.324 ±0.012	0.41 ±0.010	0.017 ±0.001
Daryai Nala	ND	0.401 ±0.012	ND	0.824 ±0.024	ND	0.790 ± 0.023	0.533 ±0.016	0.257 ±0.024	0.39 ±0.004	ND
Nehru Vihar	ND	0.339 ±0.010	ND	ND	ND	0.300 ±0.009	0.179 ±0.005	0.346 ±0.003	0.24 ±0.008	0.015 ±0.001

Limit for irrigation according to FAO (2 mg L^−1^).^22^

The concentration of heavy metals which lies above permissible limits (FAO) arc indicated with bold numeric values.

Abbreviation: ND, not detected.

### Cadmium in the Najafgarh drain

As per FAO guidelines, the threshold value of Cd in wastewater is 0.01 mg L^−1^.^1^ Cadmium remained undetected throughout the two-year sampling period *(Table 7).* It was detected in the wastewater collected from Punjabi Bagh drain sample in February 2013.

**Table 7:** Cadmium Concentrations in Wastewater at Across Sampling Sites of the Najafgarh Drain

	**Cadmium**									

**Sampling Sites**	**Jul. 2012**	**Sep. 2012**	**Dec. 2012**	**Feb. 2013**	**Apr. 2013**	**Jun. 2013**	**Aug. 2013**	**Oct. 2013**	**Jan. 2014**	**Mar. 2014**
Nilothi	ND	ND	ND	ND	ND	ND	ND	ND	ND	ND
Keshopur	ND	ND	ND	ND	ND	ND	ND	ND	ND	ND
Punjabi Bagh	ND	ND	ND	**0.075 ±0.002**	ND	ND	ND	ND	ND	ND
Daryai Nala	ND	ND	ND	ND	ND	ND	ND	ND	ND	ND
Nehru Vihar	ND	ND	ND	ND	ND	ND	ND	ND	ND	ND

Limit for irrigation according to FAO (0.01 mg L^−1^).^22^

The concentration of heavy metals which lies above permissible limits (FAO) are indicated with bold numeric values.

Abbreviation: ND, not detected.

### Nickel in the Najafgarh drain

According to FAO guidelines, the permissible limit for Ni in wastewater suitable for irrigational usage is 0.2 mg L^−1^. ^1^ The concentration of Ni was found to be well below the permissible limit at all of the sampling sites during the two-year sampling period, except during the month of August 2013. During this period, concentrations were above the permissible limit at Nilothi, Punjabi Bagh and Nehru Vihar *(Table 8).*

**Table 8:** Nickel Concentrations in Wastewater Across Sampling Sites Along the Najafgarh Drain

	**Nickel**									

**Sampling Sites**	**Jul. 2012**	**Sep. 2012**	**Dec. 2012**	**Feb. 2013**	**Apr. 2013**	**Jun. 2013**	**Aug. 2013**	**Oct. 2013**	**Jan. 2014**	**Mar. 2014**
Nilothi	ND	ND	ND	ND	ND	0.040±0.001	**0.269±0.008**	ND	ND	0.012±0.001
Keshopur	ND	ND	ND	ND	ND	ND	0.187±0.006	ND	ND	0.003±0.000
Punjabi Bagh	ND	ND	0.060±0.001	Nd	ND	0.090±0.002	**0.596±0.018**	ND	ND	ND
Daryai Nala	ND	ND	0.032±0.001	0.082±0.002	ND	0.140±0.005	0.187±0.006	ND	ND	0091±0.010
Nehru Vihar	ND	ND	ND	0.066±0.002	ND	0.100± 0.000	**0.228±0.007**	ND	ND	ND

Limit for irrigation according to FAO (0.2 mgL^−1^).^22^

The concentration of heavy metals which lies above permissible limits (FAO) are indicated with bold numeric values.

Abbreviation: ND, not detected.

### Loha mandi drain

An extensive metal analysis study was carried out in samples collected from the Loha mandi drain because this site is very near to agricultural fields. Wastewater from the Loha mandi drain is used by farmers in this area to irrigate vegetable crops. The study revealed that the average concentration of Cu was 0.155 mg L^−1^, lower than the permissible limit of 0.2 mg L^−1^, but was above the permissible limit in three different sampling periods: December 2012 (0.58 mg L^−1^), February 2012 (0.41 mg L^−1^) and April 2013 (0.34 mg L^−1^). Chromium was detected in the months of February 2013 (0.03 mg L^−1^) and January 2014 (0.09 mg L^−1^) and remained undetected during other sampling months *(Table 9).* Cadmium was found at an elevated concentration of 0.02 mg L^−1^ and was above the permissible limit for irrigation in February 2013. During the other sampling months, Cd remained undetected in wastewater. Lead was not detected in the months of July 2012, October 2013 and January 2014, but within the permissible limit of 5.0 mg L^−1^ for the other sampling months. Zinc remained below the permissible limits throughout the sampling period. However, Ni was found to be above permissible limits in the month of August 2013 during the sampling period. The average concentrations of sodium (Na), calcium (Ca), manganese (Mn), magnesium (Mg), aluminum (Al) and iron (Fe) were 79.93 mg L^−1^, 31.48 mg L^−1^, 0.15 mg L^−1^, 21.68 mg L^−1^, 4.91 mg L^−1^and 8.37 mg L^−1^, respectively. Other metals such as arsenic, antimony, barium, mercury, molybdenum, potassium, selenium, sodium, silver and tin were not detected.

**Table 9:** Heavy Metal Concentrations in Wastewater Across Sampling Sites Along the Loha Mandi Drain

**Loha mandi drain**										

**Metals**	**Jul 2012**	**Sep 2012**	**Dec 2012**	**Feb 2013**	**Apr 2013**	**Jun 2013**	**Aug 2013**	**Oct 2013**	**Jan 2014**	**Mar 2014**
Cu	ND	ND	**0.580±0.017**	**0.410±0.012**	**0.34±0.007**	0.15±0.004	0.074±0.002	ND	ND	ND
Cr	ND	ND	ND	**0.03±0.000**	ND	ND	ND	ND	**0.09±0.030**	ND
Pb	ND	0.022±0.000	0.030±0.000	0.060±0.001	0.003±0.009	0.150± 0.004	0.471±0.014	ND	ND	0.010±0.000
Zn	ND	ND	0.830±0.024	1.110±0.033	0.37±0.010	ND	0.179±0.005	ND	ND	0.145±0.033
Cd	ND	ND	ND	**0.020 ± 0.000**	ND	ND	ND	ND	ND	ND
Ni	ND	ND	ND	ND	ND	0.070±0.002	**0.228±0.007**	0	0	0.180±0.009

The concentration of heavy metals which lies above permissible limits (FAO)^22^ are indicated with bold numeric values.

Abbreviation: ND, not detected.

## Discussion

The water quality at various sampling locations could be correlated to the anthropogenic activities around the drains. Widely observed alkalinity of the water might be due to the use of detergents by residents who inhabit the catchment area of the water bodies for washing of clothes, vehicles, and utensils. The high level of chemical oxygen demand at Daryai Nala and Nehru Vihar might be due to the presence of food and beverage industries discharging untreated wastewater into the Najafgarh drain. Certain metal concentrations were higher than FAO prescribed limits at specific sampling intervals. For example, Cu was present during the August–October 2013 sampling period, possibly because stagnant water was collected from the sampling site due to construction of pillars for the Delhi Metro (rapid transit system) and parts of the drain flow was restricted. Moreover, the absence of Cr and presence of Cu was observed in samples in September 2012. Overall, the average concentrations of Cu, Cr, Pb, Zn, Cd and Ni along the Najafgarh drain were 0.168 mg L^−1^, 0.13 mg L^−1^, 0.16 mg L^−1^, 0.43 mg L^−1^, 0 mg L^−1^ and 0.04 mg L^−1^, respectively. Punjabi Bagh was the most contaminated site. The possible reason for the above observation is the presence of an industrial cluster near Punjabi Bagh. The presence of metal-related industrial clusters might be the possible reason behind the high level of Cr in the wastewater. In spite of lower Pb concentrations, the metal could pose serious threats to the environment through slow accumulation in soil and crops. In addition, although the concentration of Zn in the wastewater was low, frequent usage of contaminated wastewater may slowly build up the concentration of Zn to hazardous levels in the agricultural fields. One possible reason for this finding is a nearby industrial area that discharges wastewater directly into the drain without any treatment.^7^ From the analysis, it was evident that concentrations of heavy metals in collected wastewater samples varied according to the season across sites. This complex matrix of heavy metals in the collected water samples presents a potential human health risk from consumption of vegetables and crops irrigated with this water.^2,7^ Table 2 presents descriptive statistics for the heavy metal concentrations in wastewater of the Najafgarh and Loha mandi drains, indicating that drain wastewater was contaminated with multiple metals. The present study found that heavy metal concentrations were higher in the post-monsoon and pre-monsoon periods, when rainfall is comparatively low. High rainfall in the monsoon period may dilute the overall concentration of heavy metals.^23^

**Figure 3:**
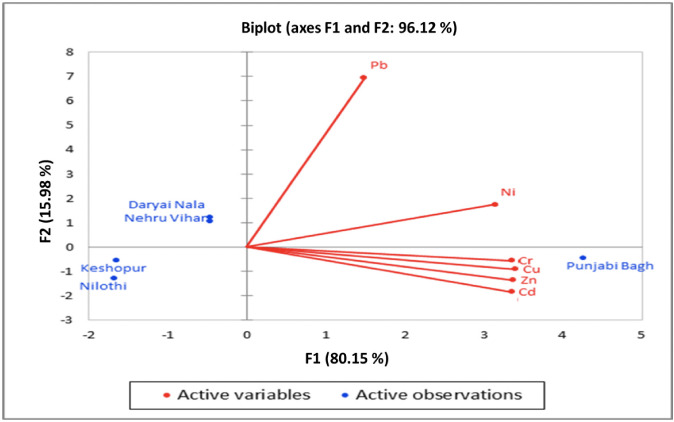
Principal component analysis performed on heavy metal data across sampling sites along the Najafgarh drain. F1 and F2 denote the factor contribution on each axis

The principal component analysis of the Najafgarh drain for the total sampling period showed that Cr, Cu, Zn and Cd had a similar source, whereas Pb and Ni had different sources. The analysis also showed that Pb and Ni were added into the drain after Punjabi Bagh, indicating that another drain joins the Najafgarh drain after Punjabi Bagh towards Nehru Vihar *(Figure 3).* This complex matrix of heavy metals in the collected water samples presents a great risk to the local population who may consume vegetables and crops irrigated with this water.

The presence of heavy metal pollutants in river and water bodies present in Delhi, India has been reported in previous studies. For example, up to 0.94 mg L^−1^, 0.008 mg L^−1^, 0.52 mg L^−1^ and 0.68 mg L^−1^ of Cr, Cu, Cd and Pb were observed in water samples from the Yamuna River, India.^24^ Water samples from multiple sampling points of the Yamuna River and drains in Delhi, India, found concentrations of Cr, Cu, and Zn above prescribed limits of 0.42 mg L^−1^, 0.64 mg L^−1^ and 2.22 mg L^−1^, respectively.^7^ The presence of heavy metals in water bodies is not limited to India. High levels of toxic heavy metals were detected in the Cross River pond (Pb: 0.455 mg L^−1^ and Zn: 0.389 mg L^−1^) compared to a tributary of the Cross River (Pb: 0.048 mg L^−1^ and Zn: 0.066 mg L^−1^) in southeastern Nigeria.^25^ High concentrations of Cr (0.110 mg L^−1^) were detected in water samples collected from the Dzindi River in South Africa.^26^ Likewise, concentrations above recommended limits for Cd (0.0150 mg L^−1^ and 0.45 mg L^−1^) were detected in collected water samples from the Nile and the Ismailia Canal (Egypt), respectively.^27,28^ Studies around the world have documented the usage of water contaminated with heavy metals for irrigational purposes.^7,15,17^ Using heavy metal-contaminated water provides an easy route for toxic metal ions to enter into the food chain via contaminated vegetable and crops.^2,15,17^ For example, multiple samples of vegetables irrigated with polluted water in the Moradabad district (India) contained high concentrations of Cr (361.50 mg kg^−1^) and Zn (258 mg kg^−1^).^29^ Similarly, high concentrations of Cu (17.20 mg kg^−1^) and Zn (120.54 mg kg^−1^) were detected in vegetables samples collected from the Baktapur district, Nepal.^30^ In another study, significant levels of Pb (0.92 mg kg^−1^) and Zn (72.7 mg kg^−1^) were detected in vegetables and crops irrigated with heavy metal-contaminated water in northern China.^31^

Consumption of contaminated vegetables and crops poses a high level of health risk to consumers. Using contaminated wastewater for irrigation not only harms human health but also deteriorates the fertility of agricultural soil.^2,7^ The presence of heavy metals in agricultural soil deteriorates the beneficial microbial population present in soil, as the hazardous and toxic nature of these metals can kill off beneficial microbes important to maintain soil fertility. The presence of multiple heavy metals, such as Cu, Cd, Pb and Zn in brown soil decreases the overall microbial biomass carbon.^32^ Similar drops in microbial biomass along with enzyme activities of these microbes (important for plant growth) were observed in the presence of agricultural soil contaminated with Zn, Cu, Ni and Cd.^33^ In another study, reduction in nitrogen fixation activity in leguminous plants was observed in the presence of heavy metals.^34^ In addition, soil polluted with toxic heavy metal ions are more acidic in nature compared to non-polluted soils.^35^ It is very much evident from the present and previous studies that the presence of heavy metals in wastewater not only deteriorates the quality of crops and vegetables, but the quality of agricultural soil as well. Appropriate treatment processes are required to decontaminate wastewater before irrigational usage.

## Conclusions

The present study revealed that most of the water samples collected from the Najafgarh and Loha mandi drains were polluted with heavy metals but showed seasonal variation in their respective concentrations. The presence of multiple metals in surface water can be attributed to effluent discharge by industries in the surrounding area. As these heavy metals might have toxic effects beyond permissible concentrations, untreated drain water is not suitable for irrigational purposes. Moreover, irrigation practices using this wastewater will further deteriorate soil quality as well as treated crops.^36,37^ Hence, adequate measures are required to remove the heavy metal load from drain water to make it suitable for irrigational purposes.
